# Rethinking cholera diagnostic test performance, interpretation, and evaluation: a field-based latent-class analysis in Bangladesh

**DOI:** 10.1016/j.lanmic.2025.101170

**Published:** 2025-10

**Authors:** Javier Perez-Saez, Taufiqur Rahman Bhuiyan, Sonia T Hegde, Ishtiakul Islam Khan, Md Taufiqul Islam, Zahid Hasan Khan, Mohammad Ashraful Amin, Juan Dent Hulse, Shakeel Ahmed, Mamunur Rashid, Rumana Rashid, Md Zakir Hossain, Ashraful Islam Khan, Firdausi Qadri, Andrew S Azman

**Affiliations:** a Geneva Centre for Emerging Viral Diseases, Geneva University Hospitals, Geneva, Switzerland; b Infectious Disease Division, International Centre for Diarrhoeal Disease Research (Bangladesh), Dhaka, Bangladesh; c Department of Epidemiology, Johns Hopkins Bloomberg School of Public Health, Baltimore, MA, USA; d Bangladesh Institute of Tropical and Infectious Diseases, Chattogram, Bangladesh; e Division of Tropical and Humanitarian Medicine, Geneva University Hospitals, Geneva, Switzerland

## Abstract

**Background:**

Accurate and reliable diagnostics, including rapid diagnostic tests (RDTs), are crucial components of cholera control programmes, although their estimated performance has varied greatly across studies. The aim of this study was to assess cholera diagnostics performance accounting for possible sources of variability, including reference assay choice and patient-level and sampling characteristics, and the implications on result interpretation and test performance evaluation.

**Methods:**

We enrolled all individuals aged 1 year and older presenting with suspected cholera seeking care at two health-care facilities in Sitakunda, Bangladesh. All participants (or, if younger than 18 years, their legal guardians) provided written informed consent and were given a short structured questionnaire on patient history and demographics, alongside a rectal swab or stool sample. All stool samples were tested with the CholKit Rapid Diagnostic Test (CholKit RDT; Incepta, Dhaka, Bangladesh), and a subset of samples (all positive RDTs and a random subset of approximately half of negative RDTs) were tested by PCR and culture. Test performance was estimated using a latent-class Bayesian framework accounting for imperfect test performance, incomplete PCR and culture testing, and time-varying changes in cholera incidence. Patient-level factor effects (including age [age 1–4 years *vs* ≥5 years] and previous antibiotic use) and sampling factor effects (season and testing delays) were estimated, and simulations were used to assess the bias in RDT performance estimates for sensitivity and specificity with 95% credible intervals (CrIs) when using traditional reference assays.

**Findings:**

Between Jan 24, 2021, and Aug 31, 2022, we enrolled 3744 individuals with suspected cholera. Of the 3744 overall participants, 1918 (51·2%) were male and 1826 (48·8%) were female; 1095 (29·2%) were aged 1–4 years and 2649 (70·8%) were 5 years and older. Among the suspected cases of cholera, 692 (18·5%) participants tested positive by the CholKit RDT. Among the RDT-positive samples, 573 (82·8%) also tested positive by PCR, and 450 (65·0%) tested positive by culture. For RDT, PCR, and culture, we estimated a sensitivity of 93·5% (95% CrI 91·3–95·4), 90·3% (88·4–92·1), and 73·7% (70·8–76·5), respectively; and a specificity of 97·3% (96·7–97·8) and 97·2% (96·6–97·8) for RDT and PCR, respectively. Culture specificity was assumed perfect at 100%. We found that younger age (1–4 years), antibiotic use, and testing delays decreased culture sensitivity, but RDT performance remained relatively constant. The RDT positive predictive value ranged from <15% in children aged 1–4 years to >80% in participants 5 years and older, varying greatly across seasons. Simulations of field trials demonstrated underestimation of RDT sensitivity in low prevalence settings when evaluated against PCR, and underestimation of specificity in high prevalence settings regardless of the reference assay.

**Interpretation:**

Our results provide potential mechanisms leading to the heterogeneous cholera RDT performance estimates in previous studies, including the use of culture as a reference assay. Across various patient and sampling characteristics, CholKit RDT had high performance in this cholera-endemic setting, supporting its use for cholera surveillance and control. Accounting for epidemiologic context is crucial both for individual-level clinical test interpretation, and for the future evaluation of diagnostics such as RDTs.

**Funding:**

The Gates Foundation.

## Introduction

Surveillance is a key pillar in the global effort to eliminate cholera, a disease caused by the bacterium *Vibrio cholerae* that still kills an estimated 95 000 people annually.^1^^,^^2^ National surveillance systems have largely relied on counting people with acute watery diarrhoea attending health facilities, which is known to have poor specificity (8·1% to 43·1% with a high probability of false positives) due to the number of other pathogenic causes of acute watery diarrhoea.^3^^,^^4^ Such difficulties in identifying the true number of cholera cases restricts our ability to rapidly detect and confirm cholera outbreaks, which in turn can have effects on population-level disease control efforts and clinical management.^5^

**Research in context**

**Evidence before this study**

Cholera remains a significant global health threat, with an estimated 95 000 deaths annually. National surveillance programmes have predominantly relied on acute watery diarrhoea case reporting, a method with poor specificity. Rapid diagnostic tests (RDTs) have been commercially available for over a decade and are recommended by the Global Taskforce for Cholera Control for surveillance and outbreak detection. On Feb 1, 2025, we searched PubMed using the following search terms:

("cholera"[Title/Abstract] OR "vibrio cholerae"[Title/Abstract]) AND ("rapid diagnostic test"[Title/Abstract] OR "rapid test"[Title/Abstract] OR "point of care"[Title/Abstract] OR "POC test"[Title/Abstract] OR "diagnostic test"[Title/Abstract] OR "diagnostic assay"[Title/Abstract]) with no restrictions on language and no restriction on the lower time bound. In total 104 studies were retrieved of which 48 were considered. RDT performance has varied widely in field evaluations, with sensitivity and specificity ranging from 66% to 100% and 47% to 96%, respectively. Such variability may be attributed to differences in reference assays (culture or PCR), patient characteristics, and sampling conditions. Despite this, a comprehensive evaluation of RDT performance accounting for these factors in a field setting has been lacking.

**Added value of this study**

This study provides a robust, field-based evaluation of the CholKit Rapid Diagnostic Test (CholKit RDT) for cholera surveillance in a cholera-endemic region of Bangladesh. Using a Bayesian latent-class model, we were able to estimate the performance of RDT, culture, and PCR without assuming any test to be a perfect reference standard. Our results show high RDT sensitivity and specificity, comparable to PCR, and a sensitivity exceeding that for culture. The study also highlights how factors such as age, antibiotic use, and delays from sample collection to testing affect the sensitivity of culture, but not RDT, reinforcing the latter’s value for real-time surveillance. Importantly, we demonstrate how traditional evaluations of RDTs against imperfect reference standards such as PCR and culture can underestimate RDT performance, especially in settings of varying cholera prevalence.

**Implications of all the available evidence**

The findings support the use of RDTs such as CholKit RDT in cholera-endemic regions, particularly where laboratory confirmation via PCR or culture is unavailable or delayed. By accounting for epidemiological context, such as cholera prevalence and patient characteristics, RDTs can provide more reliable individual-level and population-level diagnosis. Furthermore, our work emphasises the need for appropriate interpretation of RDT results, recognising that diagnostic accuracy can be influenced by factors such as seasonality and age. These insights are crucial for informing public health decisions on cholera outbreak detection, surveillance, and response strategies.

To improve true cholera case detection, the Global Task Force for Cholera Control (GTFCC) published recommendations for national cholera surveillance programmes and outbreak responses in 2024,^6^ highlighting the role of rapid diagnostic tests (RDTs) in tracking cholera incidence and rapidly identifying cholera outbreaks. To help broaden the production of and trust in cholera RDTs, the Foundation for Innovative New Diagnostics developed a target product profile focused on cholera RDTs for surveillance, setting minimum standards for test accuracy.^7^^,^^8^ Despite being commercially available for over a decade, RDT uptake by public health programmes has been low, in part due to a lack of consistency in product quality^9^ and unexplained variability in performance results during field studies,^10^ with a meta-analysis giving sensitivity ranging between 66% and 100% and a specificity ranging between 47% and 96%.^11^ Factors such as sample collection and processing, reference assays, and patient-level factors (including age, antibiotic use, and disease severity) might shape this wide variability in test performance estimates. While these advances in guidance on RDTs and the increased availability will hopefully lead to improved RDT use, a robust understanding of test performance is also needed to support regulatory authorities and public health officials in deciding how to appropriately use these tests in practice.

RDT evaluation relies on two primary laboratory techniques used to confirm cholera: culture and PCR.^12^ Many countries with endemic cholera do not have the capacity to use these assays systematically and these diagnostic approaches have imperfect performance. Culture, which relies on growing *V. cholerae* O1 and O139, is highly specific but has moderate to low sensitivity due to variability in the bacterial load of cases, sample storage and transport conditions, patient antibiotic use, and phage predation.^13^^,^^14^ Alternatively, PCR, which relies on gene amplification, tends to have higher sensitivity and high, although imperfect, specificity.^15^ Without a perfect gold standard reference assay, estimates of diagnostic field performance can be biased, although the parallel use of multiple imperfect diagnostic tests combined with latent-class statistical models can act as a solution and estimate the true performance of each test.^16^, ^17^, ^18^

Across pathogens, the interpretation of a diagnostic test result is known to not only depend on the accuracy of the test (sensitivity and specificity), but the pretest probability of disease,^19^ which can vary by geography, time, and population subgroups. Appropriate interpretation of cholera diagnostics, notably RDTs, is cardinal to the successful implementation of cholera control measures: for deciding whether to implement interventions, among whom they should be distributed, and the speed at which implementation should be done. Robust estimates of the positive and negative predictive values (NPVs) of cholera RDTs, and how they might vary across the year and by age group, are needed to inform practitioners how to interpret test results, thereby improving population-level decision making.

Here, we used unique study data from a cholera-endemic community in Bangladesh, where multiple diagnostic tests were performed in parallel along with detailed clinical surveillance data. We aimed to quantify cholera RDT, culture, and PCR performance in this field setting and to assess how patient-level and contextual factors affect diagnostic performance.

## Methods

### Study design and participants

We conducted enhanced clinical surveillance in the Sitakunda subdistrict of Chattogram, Bangladesh at the two public health facilities that serve as the primary sites for diarrhoeal disease and cholera surveillance for the Bangladesh Directorate General for Health Services in Sitakunda—the Sitakunda subdistrict hospital (Upazila Health Complex) and the Bangladesh Institute for Tropical Infectious Diseases.^20^ Sitakunda covers an area of approximately 500 km^2^ and has a population of 383 000 people.^21^ From Jan 24, 2021, to Aug 31, 2022, we attempted to enrol all individuals presenting with suspected cholera, aged 1 year and older, presenting with non-bloody, acute watery diarrhoea (three or more loose non-bloody stools in the 24 h preceding the visit) at the inpatient and outpatient wards of both facilities. We obtained written informed consent from all participants (or from legal guardians for those younger than 18 years); administered a short, structured questionnaire on patient demographics, antibiotic use, disease severity, and outcomes; and collected a stool or rectal swab specimen for laboratory analyses from all participants. More details on the structured questionnaire can be found in the appendix (pp 12–19).

The study protocol was approved by the Institutional Review Board of the International Centre for Diarrheal Disease Research, Bangladesh (icddr,b; reference 20110) and the Institutional Review Board of Johns Hopkins Bloomberg School of Public Health (reference IRB00014221).

### Procedures

We tested each participant’s faecal sample on site with the CholKit Rapid Diagnostic Test (Incepta, Dhaka, Bangladesh) following manufacturer instructions.^22^ Samples were then placed in Cary Blair media and on Whatman 903 filter paper for subsequent laboratory testing at icddr,b in Dhaka, Bangladesh. All RDT-positive samples were tested by conventional stool culture and end-point PCR (targeting *ctxA* and *rfb* genes, from filter paper).^15^ Briefly, for culture, stool was directly streaked onto selective taurocholate-tellurite gelatin agar plates, and plates were incubated overnight at 37°C. Colonies morphologically consistent with *V. cholerae* were tested for agglutination reactions with monoclonal antibodies specific to *V. cholerae* serovar O1 (Ogawa or Inaba) and O139. We additionally tested a random subset of approximately 50% of the RDT-negative samples by PCR. All diagnostic test results were used in the latent-class model.

### Statistical analysis

We performed univariate analyses on various participant and sampling characteristics to assess differences between those who were RDT-positive and RDT-negative. These characteristics included health centre of enrolment, age (categorised as either aged 1–4 years or aged ≥5 years), sex, antibiotic use before and during hospitalisation, dehydration status at hospitalisation, patient outcome, hospital stay duration, and season of enrolment. Statistical tests included Pearson’s χ^2^ test, Fisher’s exact test, and Wilcoxon rank-sum test, depending on the variable type.

To infer the performance of the CholKit RDT, PCR, and culture for cholera diagnosis, we developed a Bayesian modelling framework that accounted for the absence of a gold standard assay, partial testing within our sampling protocol (of RDT-negative samples, approximately half were tested with PCR and none with culture), as well as age-specific changes in the underlying proportion of cholera cases among people with acute watery diarrhoea during the study period. Sensitivity and specificity are presented with 95% credible intervals (95% CrIs). Priors for the sensitivity and specificity of each test were based on published estimates in a joint evaluation of CholKit with culture and PCR.^22^ We used the Stan programming language to draw samples from the posterior distribution.^23^ We assessed convergence both visually and through the Rhat statistic and evaluated model fit through posterior retrodictive checks of weekly test counts. Full details are given in the appendix (pp 2–5).

We assessed the effect of participant characteristics (age and antibiotic use before hospitalisation) and sampling details (season and delay from sample to laboratory testing) on test performance. We incorporated these variables as covariates in our estimates of test sensitivity and specificity, accounting for possible confounding between variables following our assumptions on the causal links between variables, which were made through directed acyclic graphs (appendix pp 3–4). For antibiotic use, we made the distinction in the main analysis between antibiotics recommended by the GTFCC for cholera treatment (those that typically do not yet have resistance and are effective),^24^ and other or no antibiotics. We conducted sensitivity analyses considering any antibiotic use, and all antibiotics that are known to be effective against *V. cholerae* regardless of known resistance patterns. A full list of covariate effects and regression equations controlling for possible confounding are given in the appendix (p 4). To obtain posterior estimates along covariate levels we post-stratified covariate-specific results using proportions in our sample (eg, the distribution among age classes for the effect of antibiotic use).

We conducted a simulation study to quantify the biases in RDT (or other diagnostic test) evaluations that might result from the choice of study setting due to differences in the underlying cholera prevalence and the imperfect nature of reference assays. We simulated test results by taking the mean estimates of test sensitivity and specificity in this study as references and performed 1000 simulations of test outcomes for 300 participants. Each set of simulations was done for values of cholera prevalence among acute watery diarrhoea cases ranging from 1% to 99%. We covered this wide range of possible cholera prevalence among acute watery diarrhoea cases to illustrate how this parameter can influence test evaluation biases. We then computed the corresponding estimates of RDT sensitivity and specificity taking three commonly used reference assays: PCR, culture, and a PCR–culture composite. Following previous studies, the composite reference assay considered a participant to be positive if either the PCR or culture result was positive, and negative if both PCR and culture results were negative.^18^^,^^22^

### Role of the funding source

The funder of the study had no role in study design, data collection, data analysis, data interpretation, or writing of the report.

## Results

**Figure 1:**
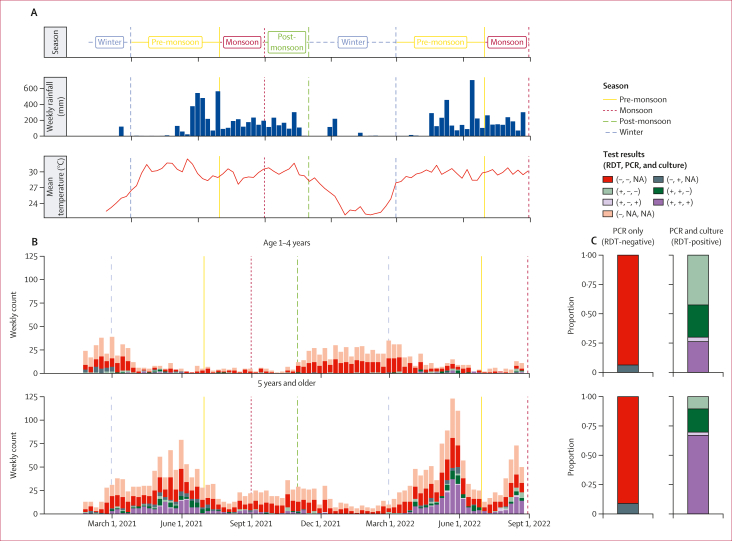
Cholera surveillance diagnostic results (A) Definition of seasons (winter, pre-monsoon, monsoon, and post-monsoon) and weather in terms of weekly precipitation and weekly mean temperature (recorded at Shah Amanat International Airport in Chattogram, Bangladesh). (B) Weekly counts by three-way category of RDT (CholKit RDT [Incepta, Dhaka, Bangladesh]), PCR, and culture test results, stratified by age category. (C) Proportion by three-way test category for samples with PCR or PCR and culture results, stratified by availability of culture results. Due to our sampling protocol, 1391 (45·6%) of 3052 RDT-negative samples were tested by PCR, and all RDT-positive samples were systematically tested by PCR and culture (right column, n=691). NA=not available. RDT=rapid diagnostic test.

From Jan 24, 2021, to Aug 31, 2022, 3744 participants were enrolled into clinical surveillance at the two health-care facilities mandated to treat cholera in the subdistrict of Sitakunda (table 1, figure 1). Of the 3744 overall participants, 1918 (51·2%) were male and 1826 (48·8%) were female; 1095 (29·2%) were aged 1–4 years and 2649 (70·8%) were 5 years and older. Among the participants with suspected cholera, 692 (18·5%) of 3744 tested positive by the CholKit RDT. There were no significant differences in RDT positivity by sex (324 [46·8%] for female participants *vs* 368 [53·2%] for male participants; p=0·26; table 1). Among the RDT-positive samples, 573 (82·8%) of 692 also tested positive by PCR, and 450 (65·0%) of 692 tested positive by culture (appendix p 6). Among 1391 (45·6%) of 3052 RDT-negative samples that were further tested by PCR, 111 (8·0%) of 1391 were PCR-positive. Positivity rates for all three tests varied significantly between age groups, with children younger than 5 years having the smallest proportion of PCR-positives among those testing positive by RDT (43 [53·1%] of 81; figure 1B, appendix p 6). The number of RDT-positive tests and PCR confirmation rates also varied strongly by season, with few RDT-positive tests being reported during the winter (November to February) and a low proportion of those being PCR-confirmed (10 [37%] of 27), compared to most RDT-positive tests being reported during the pre-monsoon period (March to June), with high PCR confirmation rates (435 [88·8%] of 490). In univariate analysis, positive RDT tests were more likely to occur among participants aged 5 years and older, during the pre-monsoon and monsoon seasons, presenting with some dehydration, without previous use of antibiotics, and with non-rice-water stool (table 1).

**Table 1:** Characteristics of enrolled participants (Jan 24, 2021–Aug 31, 2022)

	Overall (N=3744∗)	RDT-negative (n=3052)	RDT-positive (N=692)	p value†
Health-care facility				
Bangladesh Institute for Tropical Infectious Diseases	2927 (78·2%)	2298 (75·3%)	629 (90·9%)	··
Sitakunda Upazila Health Complex	817 (21·8%)	754 (24·7%)	63 (9·1%)	<0·0001
Sex				
Female	1826 (48·8%)	1502 (49·2%)	324 (46·8%)	··
Male	1918 (51·2%)	1550 (50·8%)	368 (53·2%)	0·26
Age, years	26 (21)	25 (21)	29 (18)	<0·0001
Age group				
1–4 years	1095 (29·2%)	1014 (33·2%)	81 (11·7%)	··
≥5 years	2649 (70·8%)	2038 (66·8%)	611 (88·3%)	<0·0001
Patient outcome				
Death	0	0	0	··
Discharged	3604 (96·3%)	2935 (96·2%)	669 (96·7%)	··
Transferred	13 (0·3%)	9 (0·3%)	4 (0·6%)	··
Left before being discharged	127 (3·4%)	108 (3·5%)	19 (2·7%)	0·29
Reported GTFCC-recommended antibiotic use 24 h before hospital visit‡				
0	2091 (55·8%)	1596 (52·3%)	495 (71·5%)	··
1	1640 (43·8%)	1444 (47·3%)	196 (28·3%)	··
>1	13 (0·3%)	12 (0·4%)	1 (0·1%)	<0·0001
Prescribed antibiotic use during hospital visit				
0	131 (3·5%)	118 (3·9%)	13 (1·9%)	··
1	3214 (85·8%)	2577 (84·4%)	637 (92·1%)	··
>1	399 (10·7%)	357 (11·7%)	42 (6·1%)	<0·0001
Dehydration status				
No signs	12 (0·3%)	10 (0·3%)	2 (0·3%)	··
Some	3535 (94·4%)	3015 (98·8%)	520 (75·1%)	··
Severe	196 (5·2%)	26 (0·9%)	170 (24·6%)	··
Unknown	1 (<0·1%)	1 (<0·1%)	0	<0·0001
Stool consistency at enrolment§				
Rice-water stool	265 (7·1%)	27 (0·9%)	238 (34·4%)	··
Watery stool (non-rice-water)	3475 (93%)	3022 (99·0%)	453 (65·5%)	··
Mucous	2 (0·053%)	2 (0·1%)	0	··
Semi-solid	2 (0·053%)	1 (<0·1%)	1 (0·1%)	<0·0001
Duration of hospital stay, days¶	1·9 (2·2)	1·8 (1·7)	2·1 (3·6)	··
Unknown	8	6	2	<0·0001
Season				
Pre-monsoon (March–June)	2038 (54·4%)	1547 (50·7%)	491 (71·0%)	··
Monsoon (July–August)	615 (16·4%)	464 (15·2%)	151 (21·8%)	··
Post-monsoon (September–October)	237 (6·3%)	214 (7·0%)	23 (3·3%)	··
Winter (November–February)	854 (22·8%)	827 (27·1%)	27 (3·9%)	<0·0001

Race or ethnicity data were not collected. Data are n (%) or mean (SD).

∗ No individuals meeting the suspected case definition refused to participate.

† Pearson’s χ^2^ test (on categorical variables with any expected cell count less than five); Wilcoxon rank-sum test on continuous variables; Fisher’s exact test (on categorical variables with all expected cell counts ≥5).

‡ Based on recommended antibiotic classes by the GTFCC (fluoroquinolones, macrolides, and tetracyclines).^24^

§ Rice-water stool refers to stool that is pale, greyish-white, or of a milky fluid appearance, while non-rice-water stool refers to stool that is more opaque, and yellowish or greenish in colour.

¶ The duration of hospital stay was only recorded for inpatients. GTFCC=Global Task Force for Cholera Control. RDT=rapid diagnostic test.

**Figure 2:**
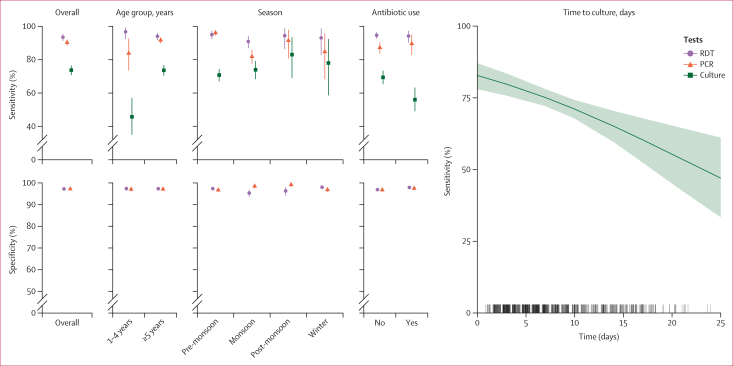
Cholera diagnostic test performance overall and by individual-specific and setting-specific factors Post-stratified estimates for RDT, PCR, and culture sensitivity and specificity, both overall and along each discrete covariate strata (age, season, and antibiotic use), and continuous covariate values (time to culture). Circles, triangles, and squares indicate the mean estimate for each test and bars indicate the 95% CrIs for categorical variables. Shaded areas indicate 95% CrIs for continuous variables. Culture specificity was assumed to be 100%. CrI= credible interval. RDT=rapid diagnostic test.

After accounting for time-varying cholera incidence rates and partial testing, we estimated a sensitivity of 93·5% (95% CrI 91·3–95·4) for RDT, 90·3% (88·4–92·1) for PCR, and 73·7% (70·8–76·5) for culture, and a specificity of 97·3% (95% CrI 96·7–97·8) for RDT, and 97·2% (96·6–97·8) for PCR (culture specificity was assumed to be 100%, following convention; table 2, figure 2). However, these mean estimates masked significant variations across patient and sampling characteristics. In regression models that account for possible confounding (appendix p 4), we found significant effects of age group on the sensitivity of nearly all three tests with the odds of testing positive among those with true cholera being significantly higher for those aged 5 years and older for PCR and culture (RDT odds ratio 3·2 [95% CrI 0·76–8·45]; PCR 0·50 [0·22–1·00]; culture 0·32 [0·19–0·51]). In addition, antibiotic use and time to sample processing had a significant effect on culture sensitivity (odds ratios for all covariates are shown in the appendix p 7). The sensitivity of culture and PCR was lower for children younger than 5 years, especially for culture where the post-stratified mean was 43·0% (95% CrI 31·7–54·9) compared with 72·3% (95% CrI 68·8–75·6) for those older than 5 years. Culture sensitivity was also lower among those reporting to have taken a GTFCC-recommended antibiotic (56·0% [95% CrI 49·1–63·3]) compared with those who did not (69·4% [65·3–73·5]), and by the delay from sample collection to testing. Sensitivity of culture reduced from 82·8% (95% CrI 78·0–87·1) when performed on the day of collection, to 65·1% (59·6–70·5) after 2 weeks, to 53·7% (42·7–64·5) after 3 weeks. Finally, the strongest seasonal differences were between PCR sensitivity in the pre-monsoon and the monsoon periods (96·1% [95% CrI 94·4–97·6] *vs* 81·9% [77·6–85·8]). We found less pronounced variations in test specificity, with only season having a significant effect on RDT, but estimates were consistently found to be above 95%, with the lowest values during the monsoon period.

**Table 2:** Cholkit RDT performance

	Reference PCR results, n		Discordant RDT results, n		Performance estimates	
	Positive	Negative	Positive	Negative	Unadjusted estimate (95% CI)	Model-adjusted estimate (95% CrI)
Sensitivity	684	··	··	11	83·8% (80·8–86·3)	93·5% (91·3–95·4)
Specificity	··	1398	118	··	91·6% (90·0–92·9)	97·3% (96·7–97·8)

Unadjusted estimates are computed using PCR as gold standard, as culture was only performed on RDT-positive samples. Model-adjusted estimates account for imperfect test performance and changes in underlying cholera incidence. CIs for the unadjusted estimates correspond to 95% Wilson binomial CIs. CrI=credible interval. RDT=rapid diagnostic test.

**Figure 3:**
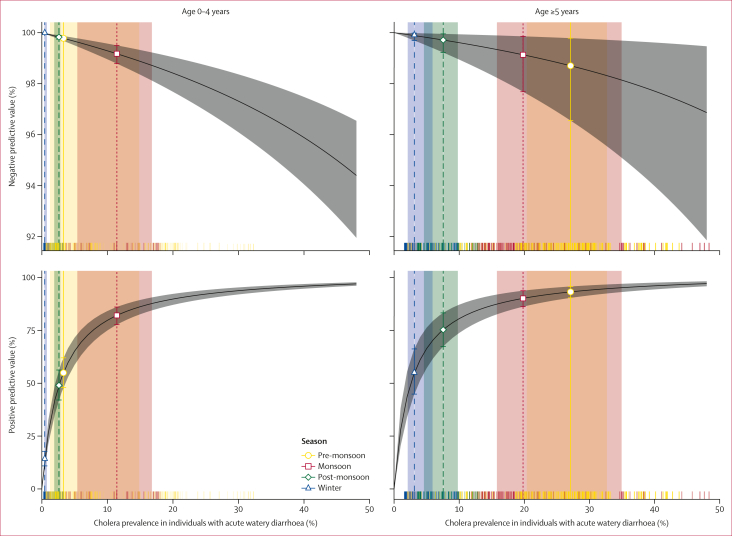
RDT negative and positive predictive values in study Negative predictive values and positive predictive values for CholKit RDTs across age groups and seasons in Sitakunda, Bangladesh. Black lines indicate the negative predictive values and positive predictive values computed at the overall mean estimates of RDT sensitivity and specificity (shown in table 2). Grey shaded areas indicate the 95% CrIs. Rug plots indicate the estimated prevalence of cholera among individuals with acute watery diarrhoea at days with at least one incidence of acute watery diarrhoea by age category during the study period. Vertical lines indicate the median cholera prevalence among those presenting with acute watery diarrhoea in each season and by age category, and dots indicate the corresponding mean negative predictive values and positive predictive values, and error bars show the corresponding 95% CrIs. CrI=credible interval. RDT=rapid diagnostic test.

Although we found that participant-level and sampling factors might affect test performance, changes in the underlying prevalence of cholera among those tested who had acute watery diarrhoea also had important implications for RDT result interpretation. Accounting for age-specific changes in seasonal cholera prevalence among individuals with acute watery diarrhoea, as well as variations in RDT performance, illustrated that the NPV of RDT was consistently high in our study (above 98%), but that the positive predictive value (PPV) varied strongly (figure 3). For children younger than 5 years, the PPV was lowest during the winter at only 14·0% (95% CrI 10·9–17·8) when the mean cholera prevalence among individuals with acute watery diarrhoea was lowest (0·45%), and highest during the monsoon period at 82·1% (77·7–86·1) when the mean cholera prevalence was 11·5%. The PPV was consistently higher in the older age class, ranging from 54·8% (44·9–66·2) in the winter with 3·1% cholera prevalence, to 93·2% (90·4–95·8) in the pre-monsoon period when mean prevalence was 27·1%. Due to lower sensitivity, the NPV of culture was systematically lower than that of RDT, including an average NPV of less than 82·5% (79·3–86·3) during the pre-monsoon season among participants aged 5 years and older (appendix p 9). Delayed culture testing also affected the NPV of culture during the pre-monsoon season, with an NPV of 95·0%, decreasing to 87·0% when samples were tested immediately compared with 3 weeks after collection.

**Figure 4:**
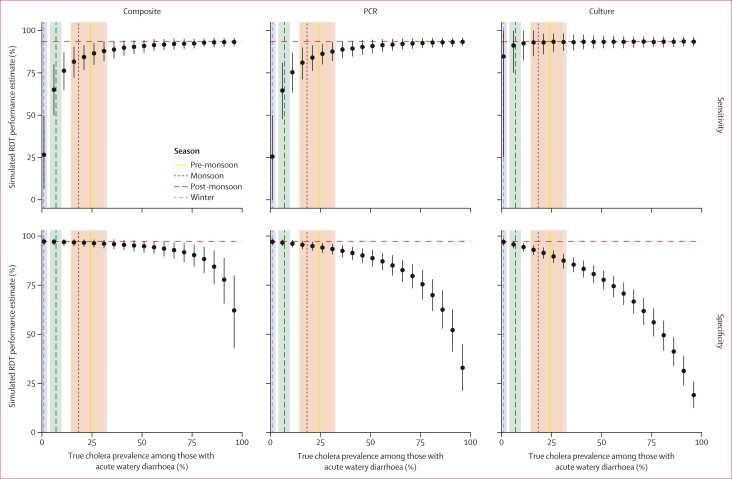
Simulations of bias in RDT evaluation due to the imperfect nature of PCR and culture RDT performance was evaluated using 1000 simulations of test outcomes for 300 participants when considering three different reference assay definitions across a range of true cholera frequencies (1% to 99%). Horizontal red dashed lines indicate the true RDT specificity and specificity values used for simulations. Alignment along the horizontal line indicates that the estimated performance aligns with that used for simulations. Dots indicate the mean and bars the 95% quantile intervals of the simulated test sensitivity or specificity values. Vertical bands represent the IQR of weekly cholera prevalence among individuals with acute watery diarrhoea within each season, with the median shown as a vertical dashed line. RDT=rapid diagnostic test.

Through simulated field evaluations, we found that RDT performance where PCR, culture, or a combination of both are used as reference standards might be severely under-estimated, and that the magnitude of the bias depends on the underlying cholera prevalence among people presenting with acute watery diarrhoea (figure 4). Underestimation of sensitivity is greatest when cholera prevalence is low and PCR or the combination of PCR and culture are used as reference standards. For example, under monsoon-season conditions (median cholera prevalence of 18% [IQR 15–32]), RDT sensitivity estimates would be less than 85% when compared to PCR or composite PCR–culture, which is lower than the true value of 93·4% used in the simulations. By contrast, the negative bias in estimates of RDT specificity increases with increasing cholera prevalence among acute watery diarrhoea, especially when using culture as the reference standard. For example, under monsoon-season conditions the estimates of RDT specificity would be less than 92% when compared to culture, which is lower than the true value of 97·3%; using the composite of PCR and culture as the reference assay would have yielded the best estimate just below the true value of 97·3%. Given a cholera prevalence among individuals presenting with acute watery diarrhoea of 75%, as is often observed in outbreak situations,^25^ RDT specificity would be greatly under-estimated when using culture (mean RDT specificity estimate of 56·1% [48·9–63·2]) or PCR (75·5% [67·8–82·7]) as reference assays, with the combination of PCR and culture yielding more accurate, although still under-estimated, results (90·4% [84·6–95·8]).

## Discussion

Through a direct comparison of rapid and traditional cholera diagnostic tests in a cholera-endemic field setting, we have presented further data on test performance and the factors that can affect this. We found that the CholKit RDT has high sensitivity (>90%) and specificity (>95%), and illustrate how various patient-specific and setting-specific factors, such as age, season, antibiotic use, time to testing, and underlying disease prevalence, can shape not only test performance, but test result interpretation at the individual level. Through this, we have shown that diagnostic field evaluations can lead to under-estimation of the sensitivity and specificity of new tests due to the imperfect nature of reference assays and that the magnitude of bias depends on the epidemiological setting.

Although the use and availability of cholera RDTs have increased in the last decade,^6^^,^^26^ concerns of product quality and variability in field performance have hindered their wide-scale deployment. Our results suggest good performance of RDTs such as CholKit in an endemic setting, similar in performance to PCR and consistent with previously published estimates.^22^ With comparatively little variability across the different patient-specific and setting-specific factors observed in our study, these results support the use of RDTs within cholera surveillance programmes and for probable outbreak detection, particularly when confirmatory testing is inaccessible. A meta-analysis of the most widely used RDT (Crystal VC, Arkay, Gujarat, India), demonstrated a pooled sensitivity of 91% (95% CI 86–95) and specificity of 75% (95% CI 69–81%), with most studies using culture as the reference standard.^11^ Given our findings and similarities in the CholKit and Crystal VC RDTs, we expect that the true specificity, and perhaps the sensitivity, of the Crystal VC test is higher than reported in the meta-analysis.

Culture has historically served as the reference diagnostic test for confirming cholera, declaring outbreaks, and evaluating novel diagnostics. Our findings add to the existing evidence that culture sensitivity is moderate to low (<75%),^22^ with drastically reduced sensitivity among children younger than 5 years (<45%), which has not, to our knowledge, previously been documented.^11^ As our age-specific estimates control for antibiotic use, lower bacterial concentrations or different co-circulating pathogens (which could interfere with the culturability of *V. cholerae* in vitro) might render culture less sensitive among children in this setting. This differential sensitivity of culture could have consequences on our interpretation of results from many seminal studies, including trials of oral cholera vaccines that used culture confirmation as the endpoint.^27^ Our analysis further confirms that, in addition to age, antibiotic use before visiting a health-care facility greatly reduces culture sensitivity (by approximately 13 percentage points).^14^^,^^15^^,^^18^ Importantly, we find that delayed culture testing might strongly increase false negative rates, with NPVs decreasing from 95·0% to 87·0% in a typical pre-monsoon season when testing samples 3 weeks after collection. These results suggest that the timely analysis of samples, patient age, and previous antibiotic use are all key for using and interpreting culture as a confirmatory assay, and particular care should be given to ruling out cholera with negative tests in many situations, in contrast to recommendations by the GTFCC.^6^

While our study was not powered to detect differences in test performance between seasons, our adjusted point estimates suggest the accuracy of cholera diagnostics might vary throughout the year. We observed reduced sensitivity of culture during pre-monsoon (high prevalence) season compared with PCR and RDT, and relatively high culture, PCR, and RDT sensitivity during post-monsoon season. Although age and antibiotic use could change seasonally, these variables were controlled for in our model, and other factors could explain differences in performance by season. Notably, the prevalence of other enteric organisms (both pathogenic and commensal) varies seasonally, and co-infection with other pathogens could inhibit concentrations and growth of *V. cholerae* O1.^28^ Lytic bacteriophages, which have appeared to have a seasonal pattern in Bangladesh, could decrease the sensitivity of diagnostics.^13^ Lastly, exposure pathways to *V. cholerae* O1 might vary seasonally due to changes in the availability and quality of water supplies,^29^ which could in turn affect the infectious dose, disease severity, and resulting concentration of bacteria in the stool. As previously shown, the ratio of symptomatic-to-asymptomatic infections changes across seasons, which could be explained by time-dependent exposure routes and infectious dose.^20^

At the individual level, our results highlight the need to account for the underlying cholera prevalence when interpreting test outcomes. The probability of true disease varies largely in time and between age classes,^25^ and as a result, the probability that a positive test result is a true positive (PPV) had a large range, from less than 15% during the winter season (<1% cholera prevalence among those with acute watery diarrhoea) among children younger than 5 years to over 90% during the pre-monsoon season (approximately 25% cholera prevalence) among those aged 5 years and older. The context-specific PPV of cholera RDTs, in some situations where the prevalence of cholera among suspected cases is high, could allow us to have high confidence in positive test results for both outbreak alerts and individual-level diagnoses, a consideration for future global surveillance recommendations.^6^

The context in which an RDT (or another new diagnostic test) is evaluated matters. The prevalence of cholera among suspected cases is a key variable when evaluating RDT performance due to the imperfect nature of reference assays. Simulations show that RDT specificity will tend to be underestimated when evaluated against culture and PCR in outbreak settings when cholera prevalence among those presenting with acute watery diarrhoea is high (eg, ≥50%), and sensitivity will tend to be underestimated when cholera prevalence is low (eg, <25%). Use of a composite PCR–culture reference assay might mitigate these biases, but does not correct for them completely. These results stress the importance of accounting for the setting and study population in which RDTs are evaluated, and the value of latent-class models to draw inference in the absence of perfect reference assays. These considerations are not limited to the evaluation of cholera RDTs, and similar qualitative patterns should be expected in any test evaluation against an imperfect reference assay.

The strength of this study is its large sample size and our joint inference of test performance accounting for imperfect tests, partial testing, and the underlying time-varying incidence of cholera. However, this study also comes with some limitations. Firstly, we did not have complete diagnostic test results for all samples, particularly for RDT-negative samples, among which no culture results were available. Although we used a statistical model to help address this partial testing,^20^ it is imperfect and could influence our inference on RDT specificity. Future studies with complete testing in cholera-endemic settings would help validate our findings. Second, because our study was conducted in an endemic setting, our overall RDT performance results and those stratified by age and season might not be generalisable to other settings. Third, we cannot exclude unmeasured confounding and model misspecification bias in our causal estimates of the effect of sample and participant covariates on diagnostic test performance. However, we provide a clear layout of our causal assumptions in the form of a directed acyclic graph (appendix p 4) which could serve as the basis for future investigations on the modifying factors of cholera test performance in field settings. Finally, the antibiotic use data in this study is based on self-reported data (and, when possible, confirmed by clinicians), which probably underestimates true antibiotic consumption, possibly leading to underestimation of the effect of antibiotic use on test performance.

This work supports the use of RDTs in cholera surveillance and an improved methodological framework for evaluating the field performance of cholera diagnostic tests. Although not a definitive diagnostic alone, if interpreted correctly RDTs can offer diagnostic support at the individual level, and can play a crucial role in surveillance for cholera control and elimination programmes. Both the accuracy and interpretation of a test are important for the rational use of diagnostics, and the GTFCC should consider the costs and benefits of context-specific recommendations, including by age and season. Given the imperfect performance of available reference assays, field evaluations should use multiple tests and latent-class models to reduce potential biases and simultaneously assess the variability of estimates across a suite of patient-specific and setting-specific factors. With the prevalent abuse of antibiotics, we need to reconsider how negative culture results are interpreted. Together, these results emphasise how dependent our assessment of cholera diagnostics is on context, and we urge policy makers to tailor recommendations appropriately in order to make progress on the pathway towards cholera elimination.

## Data sharing

Code and data to reproduce analyses from this paper are available online.

## Declaration of interests

We declare no competing interests.
