# Supplementary material for: Rethinking cholera diagnostic test performance, interpretation, and evaluation: a field-based latent-class analysis in Bangladesh
Source: Lancet Microbe. 2025 Oct;6(10):None. doi: 10.1016/j.lanmic.2025.101170 (PMC12510910; doi:10.1016/j.lanmic.2025.101170)
Supplement: Supplementary appendix [file mmc1.pdf]

THE LANCET
Microbe

Supplementary appendix

This appendix formed part of the original submission and has been peer reviewed.
We post it as supplied by the authors.

Supplement to: Perez-Saez J, Rahman Bhuiyan T, Hegde ST, et al. Rethinking cholera diagnostic test performance, interpretation, and evaluation: a field-based latent-class analysis in Bangladesh. *Lancet Microbe* 2025. https://doi.org/10.1016/j.lanmic.2025.101170

Supplementary material - Rethinking cholera diagnostic test performance, interpretation and evaluation: a field-based latent-class analysis in Bangladesh

Javier Perez-Saez, Taufiqur Rahman Bhuiyan, Sonia T Hegde, Ishtiakul Islam Khan, Md Taufiqul Islam, Zahid Hasan Khan, Mohammad Ashraful Amin, Juan Dent Hulse, Shakeel Ahmed, Mamunur Rashid, Rumana Rashid, Md Zakir Hossain, Ashraful Islam Khan, Firdausi Qadri, Andrew S Azman

Contents

S1 Modeling framework **S2**

S1.1 Latent class model . . . S2

S1.2 Participant and sampling effects on test performance . . . S3

S1.3 Correcting for possible confounding . . . S3

S2 Supplementary Figures **S6**

S2.1 Figure S2: Venn diagrams of RDT, PCR and culture results by age . . . S6

S2.2 Figure S3: Inferred effect sizes . . . S7

S2.3 Figure S4: Post-stratified estimates by age category . . . S7

S2.4 Figure S5: Sensitivity analysis on antibiotics definition . . . S8

S2.5 Figure S6: NPV and PPV for culture only . . . S9

S2.6 Figure S7: Posterior retrodictive checks . . . S10

S3 Supplementary Tables **S11**

S3.1 Age distribution of study participants . . . S11

S3.2 Antibiotic use among study participants . . . S11

S3.3 Antibiotic class among participants reporting antibiotic use . . . S12

S4 Structured questionnaire **S12**

S1 Modeling framework

The aim of the modeling framework was to estimate the infer the performance of Cholkit RDT, PCR and culture as well as the impact of patient-level characteristics and sampling factors. To do so we develop a latent class model that accounts for imperfect tests, changes in underlying cholera prevalence among AWD cases, and the specificity of our sampling protocol. In this model, the latent categorical variable is the true cholera state of each participant that can be either present or absent. We also account for possible confounding between covariates to estimate the causal effects of sample and patient characteristics on diagnostic test performance (sensitivity and specificity).

S1.1 Latent class model

S1.1.1 Probability of true cholera

For each enrolled patient presenting with AWD we model the prior probability $\phi_i(t)$ of having cholera. This probability accounts both for the estimate of the time-varying changes in the age-class specific population-level cholera prevalence among AWD case, $\rho_{a_i}(t)$, where a_i is the age class of patient i, and participant-level characteristics:

$$\text{logit}(\phi_i(t)) = \text{logit}(\rho_{a_i}(t)) + \boldsymbol{\beta}_{chol}\boldsymbol{X}_i + \gamma T(t),$$

where matrix $\boldsymbol{X}_i$ encodes participant characteristics, here age (categorical: \${$0-4, 5+$}\$) and dehydration status at admission, and $T(t)$ is the mean weekly temperature as measured in the Shah Amanata International Airport in Chattogram.

We then connect the results of RDT, PCR and culture to the patient-level probability of having cholera through the likelihood of observing test results given the underling state.

S1.1.2 Accounting for multiple tests

The surveillance scheme implemented in the study is described in details in Hegde, Khan, Perez-Saez, et al. [1] Briefly, all suspected cholera cases were tested with RDT¸ around half of RDT-negatives were tested with PCR, and all RDT-positives were tested with PCR and culture.

If all test test had been performed, the likelihood of the data would correspond to a multinomial distribution:

$$\left[n_{\{-,-,-\}}, n_{\{-,-,+\}}, n_{\{-,+,-\}}, n_{\{-,+,+\}}, n_{\{+,-,-\}}, n_{\{+,-,+\}}, n_{\{+,+,-\}}, n_{\{+,+,+\}}\right] \sim \\ multinomial(p_{\{-,-,-\}}, p_{\{-,-,+\}}, p_{\{-,+,-\}}, p_{\{-,+,+\}}, p_{\{+,-,-\}}, p_{\{+,-,+\}}, p_{\{+,+,-\}}, p_{\{+,+,+\}}),$$

where signs in brackets indicate the result of RDT, PCR and culture respectively (e.g., $\{-,+,-\}$ indicates a negative RDT, a positive PCR and a negative culture result). The vector p is the probability of a test outcome accounting both for the probability of cholera ϕ (which is be participant and time-specific as detailed above), and test sensitivity θ^+ and specificity θ^-:

$$
\begin{aligned}
p_{\{-,-,-\}} =&(1-\theta_1^+)(1-\theta_2^+)(1-\theta_3^+)\phi + \theta_1^-\theta_2^-\theta_3^-(1-\phi),\\
p_{\{-,-,+\}} =&(1-\theta_1^+)(1-\theta_2^+)\theta_3^+\phi + \theta_1^-\theta_2^-(1-\theta_3^-)(1-\phi),\\
p_{\{-,+,-\}} =&(1-\theta_1^+)\theta_2^+(1-\theta_3^+)\phi + \theta_1^-(1-\theta_2^-)\theta_3^-(1-\phi),\\
p_{\{-,+,+\}} =&(1-\theta_1^+)\theta_2^+\theta_3^+\phi + \theta^-(1-\theta_2^-)(1-\theta_3^-)(1-\phi),\\
p_{\{+,-,-\}} =&\theta_1^+(1-\theta_2^+)(1-\theta_3^+)\phi + (1-\theta_1^-)\theta_2^-\theta_3^-(1-\phi),\\
p_{\{+,-,+\}} =&\theta_1^+(1-\theta_2^+)\theta_3^+\phi + (1-\theta_1^-)\theta_2^-(1-\theta_3^-)(1-\phi),\\
p_{\{+,+,-\}} =&\theta_1^+\theta_2^+(1-\theta_3^+)\phi + (1-\theta_1^-)(1-\theta_2^-)\theta_3^-(1-\phi),\\
p_{\{+,+,+\}} =&\theta_1^+\theta_2^+\theta_3^+\phi + (1-\theta_1^-)(1-\theta_2^-)(1-\theta_3^-)(1-\phi),
\end{aligned}
$$

where subscripts 1,2,3 denote RDT, PCR and culture, respectively.

Due to our sampling protocol, we do not have test results of PCR and culture for certain participants. To account for partial testing, we follow our previous approach which factors in the conditional probabilities of unobserved PCR and culture conditional on the result of RDT. We refer the reader to our Hegde, Khan, Perez-Saez, et al. [1] for further details (Methods, section "Statistical analysis").

S1.2 Participant and sampling effects on test performance

We here account for the impact of participant characteristics (age, antibiotic use) and sampling (RDT batch, season, time to culture) on test performance by incorporating them as covariates in our latent class model. Specifically, we assume that the sensitivity, θ^+, and specificity, θ^-, of RDT, PCR, and culture follow a logit-linear model:

$$
\begin{aligned}
logit(\theta_j^+) &= \beta_{j,0}^+ + \boldsymbol{\beta}_j^+ \boldsymbol{\mathcal{X}}_j^+,\\
logit(\theta_j^-) &= \beta_{j,0}^- + \boldsymbol{\beta}_j^- \boldsymbol{\mathcal{X}}_j^-,
\end{aligned}
$$

where j is the test index as above (1: RDT, 2:PCR, 3:culture), $\beta_{j,0}^{+/-}$ is the intercept, $\boldsymbol{\beta}_j^{+/-}$ is the vector of covariate coefficients, and $\boldsymbol{\mathcal{X}}_j^{-/+}$ is the covariate matrix.

S1.3 Correcting for possible confounding

The relationship between participant characteristics, sampling factors and test performance may be complex and presents the challenge of estimating effects in the presence of possible confounding between factors. To address this we propose a directed acyclic graph representing our assumptions on how these measurable factors are causally connected through unmeasurable quantities which ultimately may impact the performance of RDT, PCR and culture (Figure S1).

Given each DAG and covariate, we define the minial set of covariates to control for possible confounding. We do so automatically using the dagitty package in R using the function `adjustmentSets`. The final set of equations used for each factor and covariate is given in Table S1.

To account for possible RDT batch effects, following our previous analysis we separate the modeling period into two distinct periods, one from the start of the study up to June 29th 2021, and one from June 30th to the end of the study period [1].

Table S1: Regression equations to control for possible confounding based on DAGs.

	Test characteristic	
covariates	sensitivity	specificity
**RDT**		
age	age + antibiotic use + RDT batch	age
antibiotics	antibiotic use + age + RDT batch	antibiotic use + age
batch	RDT batch + age + season	
period	season + RDT batch	season
time to culture		
**PCR**		
age	age + antibiotic use	age + RDT batch
antibiotics	antibiotic use + age	antibiotic use + age + RDT batch
batch		
period	season	season + RDT batch
time to culture		
**culture**		
age	age + antibiotic use + time to culture	
antibiotics	antibiotic use + age + time to culture	
batch		
period	season + time to culture	
time to culture	time to culture	

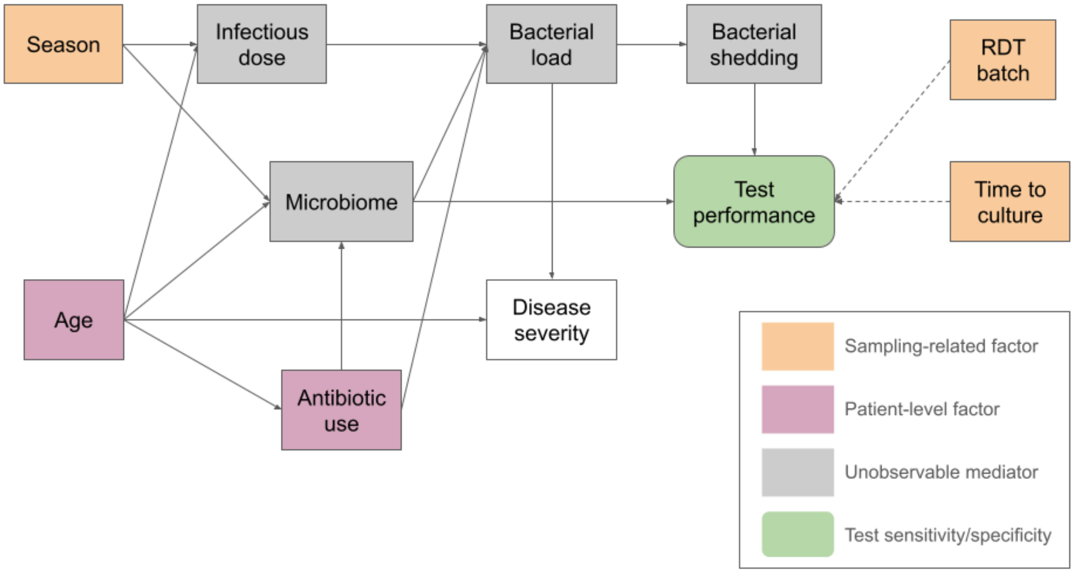

Figure S1: Directed acyclical graph of causal relations between patient-level and sampling fractors and test performance. Full arrows into test performance indicate that they affect the sensitivity of all tests. Dotted lines indicate factors that only affect certain test (RDT batch affects RDT sensitivity, and time to culture affects culture sensitivity).

S1.3.1 Priors

We use the following priors in the cholera incidence model with no differences between age classes:

$$\begin{aligned}
\beta_{chol} &\sim \mathcal{N}(0, 0.5) \\
\gamma &\sim \mathcal{N}(0, 0.5) \\
\beta^{+}_{RTD,0} &\sim \mathcal{N}(0, 0.75), \\
\beta^{+}_{PCR,0} &\sim \mathcal{N}(1.04, 0.34), \\
\beta^{+}_{culture,0} &\sim \mathcal{N}(0.89, 0.26), \\
\beta^{-}_{RTD,0} &\sim \mathcal{N}(3.48, 0.69), \\
\beta^{-}_{PCR,0} &\sim \mathcal{N}(3.48, 0.44), \\
\beta^{+/-}_{i} &\sim \mathcal{N}(0, 1),
\end{aligned}$$

,

where $\beta^{+/-}_{i,0}$ indicates the intercept for each test performance on the logit scale, $\beta^{+/-}_{i}$ the regression coefficients of the effect of covariates on test performance, and β_{chol} the regression coefficients of the probability of true cholera. By $\mathcal{N}(a, b)$ we intend an Normal distribution with mean a and variance b^2. Priors for the intercepts followed our previous work in Hegde, Khan, Perez-Saez, et al. [1] based on results in Sayeed, Islam, Hossain, et al. [2]. We chose weekly informative priors on β_{chol} and γ assuming effects of categorical covariates and standardized temperature centered on no effect (0 on the logit scale) and covering effect sizes with an approximate 95% prior interval of $+/-1.5$ on the logit scale (odd ratios in the range of 0.2-4). Similarly we chose a 0-centered prior on the regression coefficients $\beta^{+/-}_{i}$, covering effect sizes with an approximate 95% prior interval of $+/-3$ on the logit scale (odd ratios in the range of 0.05-20).

S2 Supplementary Figures

S2.1 Figure S2: Venn diagrams of RDT, PCR and culture results by age

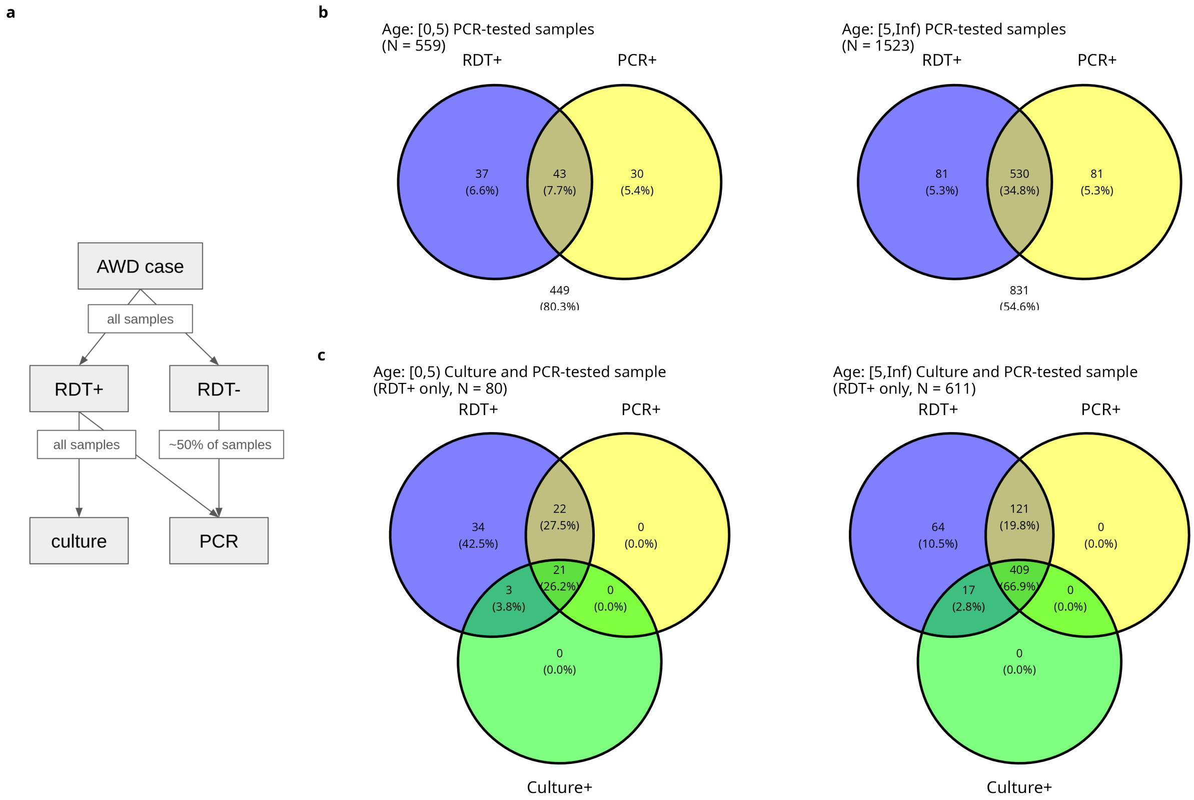

Figure S2: Raw test results. a) Study sampling scheme. All AWD cases in the study health centers were tested with RDT. All positive RDT samples were also tested with PCR and culture. Around half of RDT negative samples were tested with PCR. b) Venn diagrams of RDT and PCR test results by age class for all PCR-tested samples. c) Venn diagrams of RDT, PCR and culture test results by age class for all RDT-positive samples.

S2.2 Figure S3: Inferred effect sizes

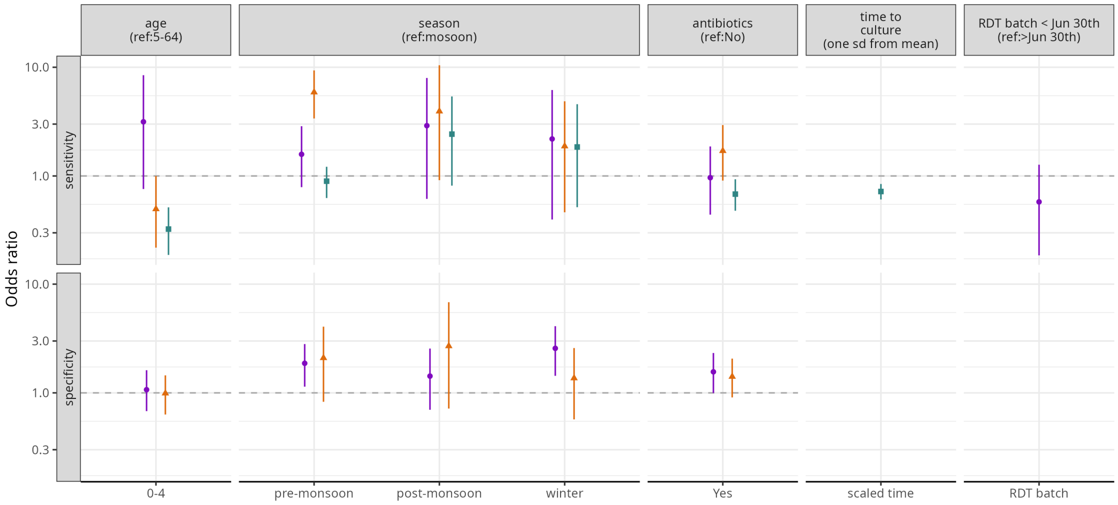

Figure S3: Covariate effect sizes on test performance. Inference of effect sizes accounts for possible confounding as described in section S2.3. Dots indicate mean of 5000 posterior Hamiltonian Monte Carlo draws, and bars the 95% CrIs. Colors and point shapes indicate diagnostic test: RDT (purple, dots), PCR (orange, triangles) or culture (green, squares).

S2.3 Figure S4: Post-stratified estimates by age category

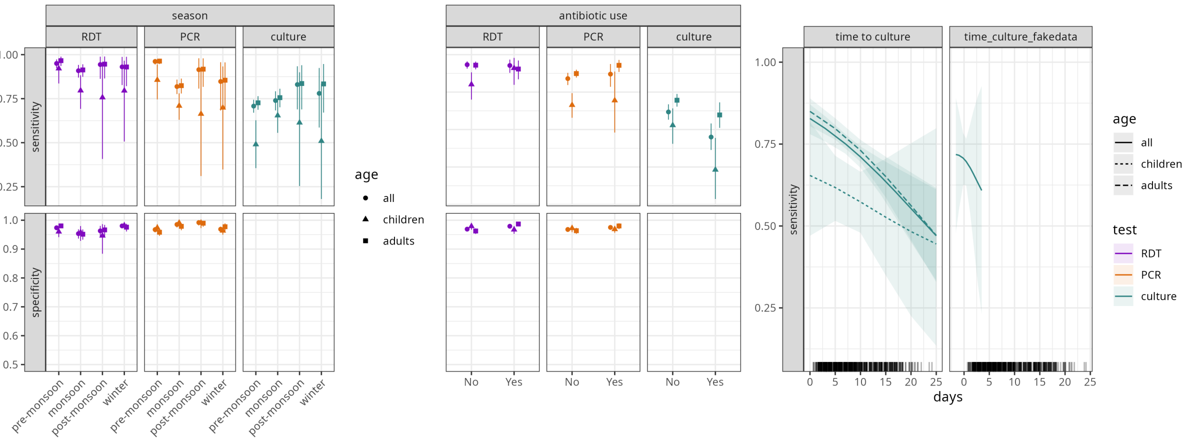

Figure S4: Post-stratified estimates by age class. Legend as in main Figure 2. Dots indicate the mean of 5000 posterior Hamiltonian Monte Carlo draws, bars the 95% CrIs for categorical variables, and shading the 95% CrIs for continous variables.

S2.4 Figure S5: Sensitivity analysis on antibiotics definition

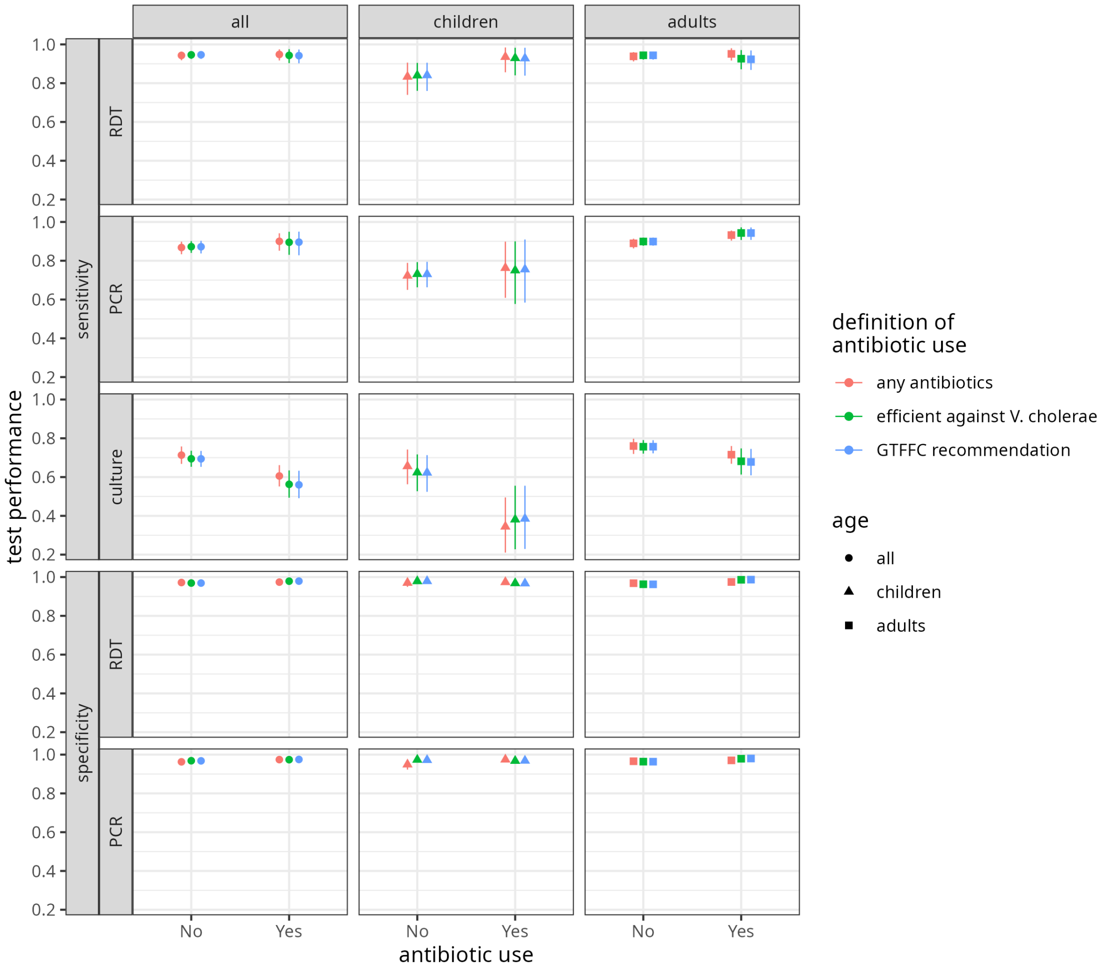

Figure S5: Post-stratified estimates for alternative definitions of antibiotic use. In addition to any antibiotic use, we grouped antibiotics either by whether they are recommended antibiotic classes by the GTFCC (tetracyclines, fluoroquinolones, and macrolides), or known to be effective against V. cholerae (fluoroquinolones, macrolides, tetracyclines, penicillins, sulfonamides, cephalosporins). We report the GTFCC-recommedended results in the main.

S2.5 Figure S6: NPV and PPV for culture only

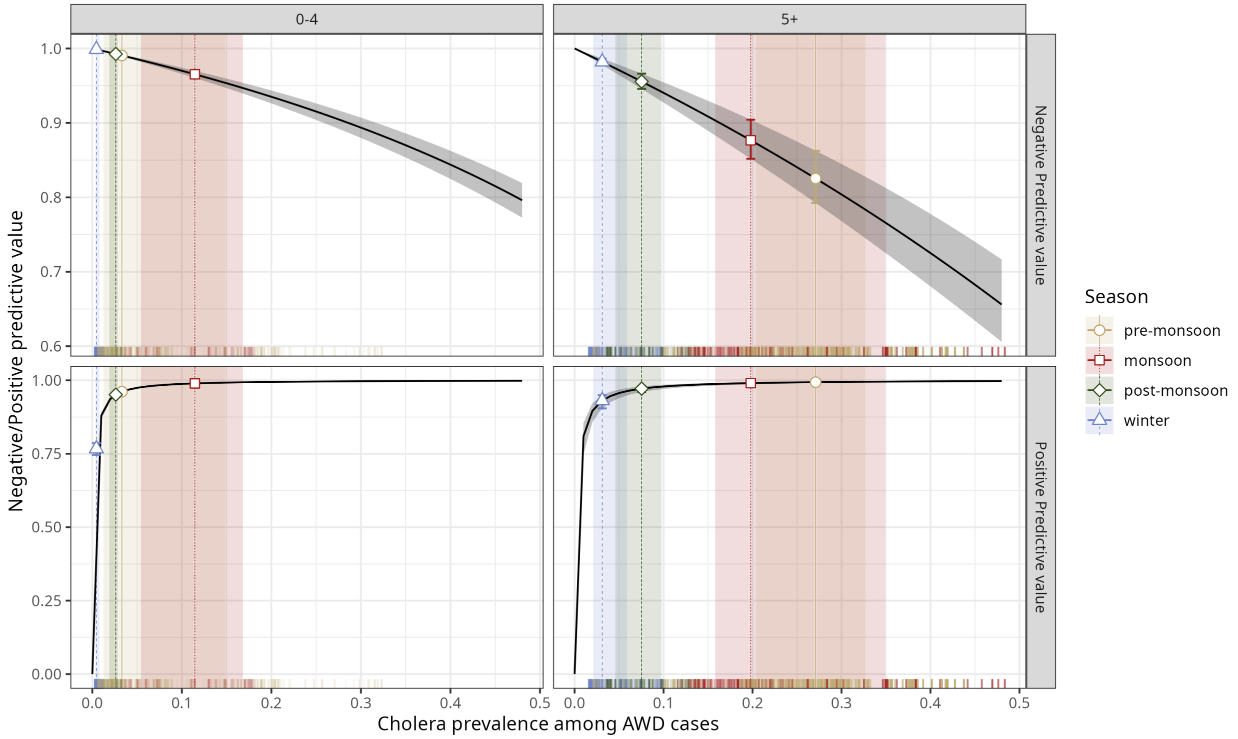

Figure S6: Negative and positive predictive values when using culture. Lines represent the mean of 5000 posterior Hamiltonian Monte Carlo draws, and shading the 95% CrIs.

S2.6 Figure S7: Posterior retrodictive checks

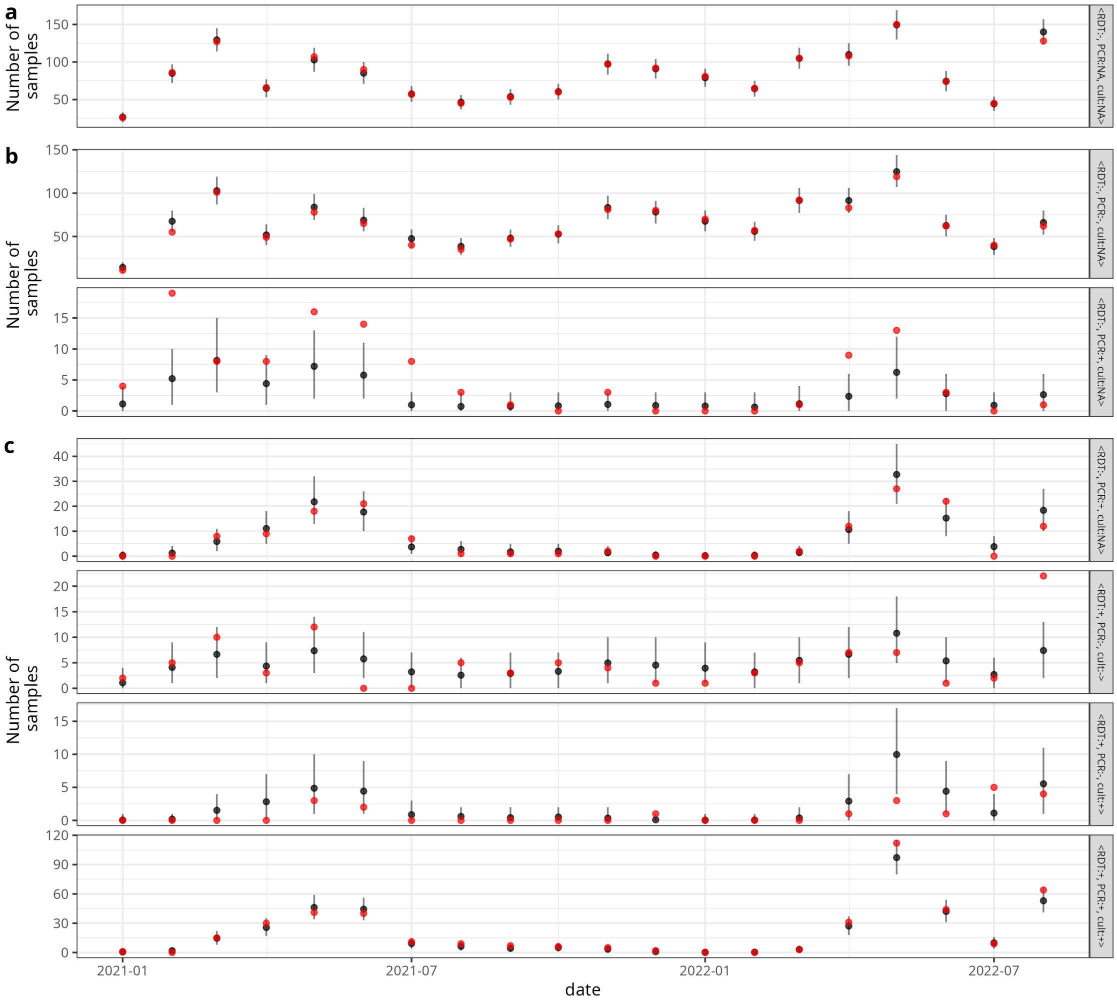

Figure S7: Posterior retrodictive checks of surveillance data. Red dots indicate monthly observed counts. Black dots indicate the mean of 5000 Hamiltonian Monte Carlo posterior draws, and error bars the 95% CrI. a) Samples with RDT negative results only. b) Samples with both RDT and PCR result, but no culture. c) RDT positive samples with PCR and culture results.

S3 Supplementary Tables

S3.1 Age distribution of study participants

Table S2: The age distribution of the study population in Sitakunda, Bangladesh.

Age category	Overall, N = 3,744	RDT-negative, N = 3,052	RDT-positive, N = 692
[0,5)	1,095 (29%)	1,014 (33%)	81 (12%)
[5,10)	125 (3.3%)	87 (2.9%)	38 (5.5%)
[10,15)	65 (1.7%)	47 (1.5%)	18 (2.6%)
[15,25)	516 (14%)	350 (11%)	166 (24%)
[25,35)	636 (17%)	495 (16%)	141 (20%)
[35,45)	487 (13%)	386 (13%)	101 (15%)
[45,55)	392 (10%)	321 (11%)	71 (10%)
[55,65)	252 (6.7%)	210 (6.9%)	42 (6.1%)
[65,75)	136 (3.6%)	113 (3.7%)	23 (3.3%)
[75,85)	36 (0.96%)	26 (0.85%)	10 (1.4%)
[85,Inf)	4 (0.11%)	3 (0.098%)	1 (0.14%)

S3.2 Antibiotic use among study participants

Table S3: Antibiotic use by RDT positivity among all study participants: those that took any type of antibiotic, those that took antibiotics recommended by the GTFCC (tetracyclines, fluoroquinolones, macrolides), and those that took antibiotics that are known to be effective against killing V. cholerae (fluoroquinolones, macrolides, tetracyclines, penicillins, sulfonamides, cephalosporins).

Type of antibiotic use	Overall, N = 3,744	RDT-negative, N = 3,052	RDT-positive, N = 692
**All reported antibiotic use 24hr prior to hospital visit**			
0	1,181 (32%)	878 (29%)	303 (44%)
1	1,855 (50%)	1,559 (51%)	296 (43%)
1+	708 (19%)	615 (20%)	93 (13%)
**Reported GTFCC recommended antibiotic use 24hr prior to hospital visit**			
0	2,091 (56%)	1,596 (52%)	495 (72%)
1	1,640 (44%)	1,444 (47%)	196 (28%)
1+	13 (0.35%)	12 (0.39%)	1 (0.14%)
**Reported effective antibiotic use 24hr prior to hospital visit**			
0	2,062 (55%)	1,569 (51%)	493 (71%)
1	1,662 (44%)	1,464 (48%)	198 (29%)
1+	20 (0.53%)	19 (0.62%)	1 (0.14%)

S3.3 Antibiotic class among participants reporting antibiotic use

Table S4: The number of instances antibiotics were taken by study participants prior to the health facility visit by antibiotic class. Tetracyclines, fluoroquinolones, and macrolides are the recommended antibiotic classes by the GTFCC. The nitromidazole antibiotic primarily taken is metronidazole.

	Age class		
Antibiotic class	less than 5	5+	Overall
Tetracycline*	1 (0.10%)	1 (0.043%)	2
Fluoroquinolone*	538 (56%)	876 (38%)	1414
Macrolide*	101 (10%)	149 (6.4%)	250
Nitroimidazole	309 (32%)	1272 (55%)	1581
Penicillin	1 (0.10%)	4 (0.17%)	5
Sulfonamide	1 (0.10%)	1 (0.043%)	2
Cephalosporin	14 (1.5%)	15 (0.65%)	29
Chloramphenicol	0 (0.0%)	1 (0.043%)	1
Total	965 (100%)	2319 (100%)	3284

S4 Structured questionnaire

We administered the following structured questionnaire to enrolled study participants at each health facility. This questionnaire asks questions about the date of patient enrollment, patient demographics, antibiotic use prior to and during hospitalization, dehydration status at hospitalization, where patients are visiting from, and patient outcomes.

Clinical Surveillance Report Form

Study ID

____

Clinical Surveillance Form is to be completed by the study staff at each healthcare facility for consenting individuals ≥1 year of age who are experiencing acute watery diarrhea (3 or more watery, non-bloody stools in the last 24 hours) in both inpatient and outpatient care. The eligibility criteria of each participating individual should be identified before completing this survey.

Unsure and Decline tick boxes are there for the field researcher to record if participants give such responses to questions, they should not be read out to participants.

Timestamp creation of form

____

Module 1: Identification (CQ1) To be filled out by the interviewer for each consenting individual >=1 year of age.

1.1 Interview Date

Please verify that the auto-generated date is correct

______________________________ (YYYY-MM-DD)

Warning: The date entered occurs in the future, please revise before continuing.

1.2 Hospital facility

◯ BITID
◯ Sitakunda Upazila Health Complex

1.3 Interviewer Name

____

1.4 Is the patient currently receiving inpatient or outpatient care?

◯ Inpatient ◯ Outpatient

1.5 What is the relationship of the individual being interviewed to the study participant?

◯ Self
◯ Guardian
◯ Relative
◯ Friend
◯ Other

Please specify the respondent's relationship to study patient

____

The following questions should be answered on behalf of the patient and not asked directly to the patient.	
Module 2: Clinical and Demographic Information (CQ1)<br>Record the following information with the patient and with the healthcare staff or diarrhea registry if needed.	
2.1 Have you had 3 or more watery, non-bloody stools in the last 24 hours?	◯ Yes<br>◯ No
2.2 How old are you (in years)?	______________________<br>(if less than 1 enter 0)
2.3 What is your sex?	◯ Male<br>◯ Female<br>◯ Decline
2.4.1 In which district is your home located?<br><br>If you start typing the first and second letters of the location in the response drop down menu, a list of possible names will appear that are spelled similarly.	
2.4.2 In which subdistrict is your home located?<br><br>If you start typing the first and second letters of the location in the response drop down menu, a list of possible names will appear that are spelled similarly.<br><br>If the address is not listed in this menu, select "Other (specify)" and record the location name in a new field that will appear below this question.<br><br>Please specify the subdistrict in which your home is located	______________________
2.4.3 In which union is your home located?<br><br>If you start typing the first and second letters of the location in the response drop down menu, a list of possible names will appear that are spelled similarly.<br><br>If the address is not listed in this menu, select "Other (specify)" and record the location name in a new field that will appear below this question.<br><br>Please specify the union in which your home is located	______________________
2.4.4 In which village is your home located? __________	

2.5 In which location did you spend the most time during the last 7 days?	◯ Same as home address<br>◯ Elsewhere<br>◯ Don't know<br>◯ Decline
2.5.1 In the last 7 days, in which district have you spent most of your time?<br><br>If you start typing the first and second letters of the location in the response drop down menu, a list of possible names will appear that are spelled similarly.	
2.5.2 In which subdistrict of [cat_time_district] did you spend most of your time in the last 7 days?<br><br>If you start typing the first and second letters of the location in the response drop down menu, a list of possible names will appear that are spelled similarly.<br><br>If the address is not listed in this menu, select "Other (specify)" and record the location name in a new field that will appear below this question.<br><br>Please specify the subdistrict in which you spent most of your time in the last 7 days	______________________________
2.5.3 In which union of [cat_time_subdistrict] did you spend most of your time in the last 7 days?<br><br>If you start typing the first and second letters of the location in the response drop down menu, a list of possible names will appear that are spelled similarly.<br><br>If the address is not listed in this menu, select "Other (specify)" and record the location name in a new field that will appear below this question.<br><br>Please specify the union in which you spent most of your time in the last 7 days	______________________________
2.5.4 In which village of this union did you spend most of your time in the last 7 days? ___________	
2.6 What date did you arrive at this health facility?	
2.7 What symptoms did you have in the 2 days prior to coming to this facility? (Check all that apply)	☐ Fever<br>☐ Chills<br>☐ Watery stool<br>☐ Nausea<br>☐ Vomiting<br>☐ Other (specify)<br>☐ Don't know<br>☐ Decline

Please specify any other symptoms you have had in the 2 days prior to coming to this facility.	________________________________
2.8 When did the first of these symptoms start?	
2.9 Did you seek care for your current symptoms elsewhere before coming to this facility (e.g., another health facility, pharmacy, traditional healer)?	☐ Nowhere, this is the first place I sought care<br>☐ Health Facility<br>☐ Pharmacy<br>☐ Traditional Healer<br>☐ Other<br>☐ Don't know<br>☐ Decline
What is the name of the health facility you visited?	________________________________
What is the name of the pharmacy you visited?	________________________________
What is the name of the traditional healer you visited?	________________________________
What is the name of the other facility you visited?	________________________________
2.10 Have you taken any medication to treat your symptoms in the 24 hours prior to your visit to this healthcare facility?<br><br>This should only include medications taken before your visit to this healthcare facility	◯ No<br>◯ Yes<br>◯ Don't know<br>◯ Decline
2.11 What is the name of the medicine you took? (Check all that apply)	☐ Metronidazole<br>☐ Ciprofloxacin<br>☐ Amoxycillin<br>☐ Azithromycin<br>☐ Penicillin<br>☐ Tetracyclin<br>☐ Cotrimoxazole<br>☐ Flucloxacillin<br>☐ Levofloxacillin<br>☐ Cefuroxime<br>☐ Cefixime<br>☐ Doxycyclin<br>☐ Chloramphenicol<br>☐ Ceftriaxone<br>☐ Loperamide (Imodium)<br>☐ Bismuth subsalicylate (Kaopectate, Pepto-Bismol)<br>☐ Paregoric (Anhydrous Morphine)<br>☐ Other (specify)<br>☐ Don't Know<br>☐ Decline
Please specify the name of the other medicine you took	________________________________

Record the following information either with the healthcare staff or from the diarrheal disease registry.	
2.12 What was the dehydration status of the patient at the time of first assessment?	◯ No signs<br>◯ Some<br>◯ Severe<br>◯ Don't know
Module 3: Sample collection (CQ1)<br>Record the following information either from the laboratory technician, the healthcare staff or from the diarrheal disease registry	
3.1 Record the sample ID<br><br>The sample ID is the ID written on the stool sample label and should be the same as the participant and record ID.	______________________
3.2 Sample collected?	◯ No<br>◯ Yes, stool sample<br>◯ Yes, rectal swab
3.3 Why was a stool sample not collected?	◯ Patient no longer in care/available for stool collection (died/transferred/discharged)<br>◯ Patient refused stool collection<br>◯ Patient unable to produce stool<br>◯ Other
Please specify why a stool sample was not collected	______________________
3.4 Sample collection date and time<br><br>Please verify that the auto-generated date is correct	______________________<br>(YYYY-MM-DD HH:MM)
3.5 Please add any relevant comments here:	______________________

Module 4: Patient outcome. Record the following information for all inpatient participants either from the patient her/himself or with the healthcare staff or from the diarrheal disease registry.

4.1 Interviewer Name	______________________________
4.2.1 Did the patient receive any antibiotics while in care?<br><br>Answer using the patient's clinical records	◯ No<br>◯ Yes<br>◯ Don't know
4.2.2 What antibiotic did the patient take?<br><br>Answer using the patient's clinical records	☐ Metronidazole<br>☐ Ciprofloxacin<br>☐ Amoxycillin<br>☐ Azithromycin<br>☐ Penicillin<br>☐ Tetracyclin<br>☐ Cotrimoxazole<br>☐ Flucloxacillin<br>☐ Levofloxacillin<br>☐ Cefuroxime<br>☐ Cefixime<br>☐ Doxycyclin<br>☐ Chloramphenicol<br>☐ Ceftriaxone<br>☐ Other<br>☐ Don't Know
Please specify which other antibiotic the patient took	______________________________
4.2.3 Were antibiotics given to the patient in this health centre before the stool sample was collected?	◯ No<br>◯ Yes<br>◯ Don't know
4.3 What was the patient outcome?	◯ Death<br>◯ Discharged<br>◯ Transferred<br>◯ Left before discharged<br>◯ Don't know
4.4 What date did this outcome occur on?<br><br>Please verify that the auto-generated date is correct	______________________________<br>(YYYY-MM-DD)
4.5 What type of facility did she/he get transferred to?	◯ Tertiary care<br>◯ Government facility<br>◯ Private facility<br>◯ Other<br>◯ Don't know

Please specify what other kind of facility the patient was transferred to	______________________________
4.6 What is the name of the facility that she/he was transferred to?	______________________________
4.7 Please add any relevant comments here:	______________________________
If data collection on this form is complete, please select "Now" to specify the current date and time.	______________________________<br>(YYYY-MM-DD HH:MM)

References

1. Hegde ST, Khan AI, Perez-Saez J, et al. Clinical surveillance systems obscure the true cholera infection burden in an endemic region. Nature medicine 2024;30:888–95.
2. Sayeed MA, Islam K, Hossain M, et al. Development of a new dipstick (Cholkit) for rapid detection of Vibrio cholerae O1 in acute watery diarrheal stools. PLoS neglected tropical diseases 2018;12:e0006286.
